# The Role of Endophytes in Combating Fungal- and Bacterial-Induced Stress in Plants

**DOI:** 10.3390/molecules27196549

**Published:** 2022-10-03

**Authors:** Manjula Muthu Narayanan, Norhayati Ahmad, Pooja Shivanand, Faizah Metali

**Affiliations:** Environmental and Life Sciences Program, Faculty of Science, Universiti Brunei Darussalam, Jalan Tungku Link, Bandar Seri Begawan BE1410, Brunei

**Keywords:** antagonism, biocontrol mechanism, endophytes, induced stress

## Abstract

Plants are subjected to multifaceted stresses that significantly jeopardize crop production. Pathogenic microbes influence biotic stress in plants, which ultimately causes annual crop loss worldwide. Although the use of pesticides and fungicides can curb the proliferation of pathogens in plants and enhance crop production, they pollute the environment and cause several health issues in humans and animals. Hence, there is a need for alternative biocontrol agents that offer an eco-friendly mode of controlling plant diseases. This review discusses fungal- and bacterial-induced stress in plants, which causes various plant diseases, and the role of biocontrol defense mechanisms, for example, the production of hydrolytic enzymes, secondary metabolites, and siderophores by stress-tolerant fungi and bacteria to combat plant pathogens. It is observed that beneficial endophytes could sustain crop production and resolve the issues regarding crop yield caused by bacterial and fungal pathogens. The collated literature review indicates that future research is necessary to identify potential biocontrol agents that can minimize the utility of synthetic pesticides and increase the tenable agricultural production.

## 1. Introduction

By 2050, there will be a considerable increase in the population of around 9.7 billion, leading to an increased requirement for food [1,2]. According to the Food and Agriculture Organization (FAO) of the United Nations, at least a 50% increase in the average agricultural food production must be achieved by 2050 [2]. Thus, a strategy is required to mitigate pre- and post-harvest crop yield losses and enhance crop production to meet population demands [1]. Plants are most vulnerable to biotic stresses caused by microbes that hinder the sustainability of agriculture, leading to global crop catastrophe [3]. Biotic stresses cause approximately a 17 to 30% decline in global crop production [3].

Fungi and bacteria are the major causative agents, which produce 70–80% of plant infections leading to universal crop calamity [3,4]. These pathogens invade the plants through the roots, stomata, or open wounds resulting from adverse weather conditions, human activities (handling tools and machinery), insects, and other vectors, and cause disease in plants by secreting either extracellular enzymes or secondary metabolites or toxins [5,6,7]. Most plant pathogenic fungi species are *Alternaria* spp., *Aspergillus* spp., *Colletotrichum* spp., *Fusarium* spp., *Phytophthora* spp., and *Pythium* spp. These species cause various infections in plants such as anthracnose, dieback, gall, powdery mildew, blight, rust, rot, wilt, and smut. Some manifestations are overgrowth, deformations, mummification, wilting, spotting, mold, and pustules [4]. For example, Aspergillus niger causes ear rot, yellow mold, and black mold in cereal grains, legumes, nuts, grapes, apricots, and onions [8] and ***Blumeria* spp.** cause powdery mildew in grasses and cereals and has symptoms of white, powdery spots or patches on the plant stems or leaves [9]. Phytopathogenic bacteria are grouped under the following genera: *Agrobacterium*, *Bacillus*, *Burkholderia*, *Clavibacter*, *Erwinia*, *Pantoea*, *Pseudomonas*, *Ralstonia*, *Streptomyces*, *Xanthomonas*, and *Xylella*. Galls and overgrowths, wilts, soft rots, scabs, cankers, leaf spots, and blights are traits caused by pathogenic bacteria [6]. For example, the first bacterial disease was anthrax caused by *Bacillus anthracis*, infecting cattle and sheep in 1876. Fire blight disease in apple and pear in addition to other fruits from the Rosaceae family was first discovered by T. J. Burrill from the University of Illinois during the period 1877–1885 [10]. This disease, which originated in North America, has now spread to 50 countries in Europe, Africa, the Middle East, and Asia. Another bacterial disease that is considered a serious threat to the tomato and pepper industries around the world is bacterial canker or ring rot, which is caused by *Clavibacter michiganensis* strains [11].

Although chemical pesticides curb biotic stress and enhance crop production, they cause adverse environmental consequences such as soil acidification and groundwater contamination, which **impede** the growth of plant roots and **destroy** the beneficial rhizosphere microbes [12]. Prolonged use of chemical pesticides causes a health risk to humans, for example, pesticides such as glyphosate, dichlorodiphenyltrichloroethane (DDT), dichlorodiphenyldichloroethylene (DDE), and dichlorodiphenyldichloroethane (DDD) cause acute poisoning and apneic seizures in humans, leading to death [12]. In addition, such use gives rise to resistive pathogenic strains such as *Aspergillus*, *Alternaria*, *Botrytis*, *Colletotrichum*, *Fusarium*, *Penicillium*, *Phytophthora*, *Verticillium*, and *Ustilago* [13]. Therefore, there is a need for an alternative natural source of plant disease management known as biocontrol. Microbes are a novel source of naturally available biocontrol agents that combat or inhibit the growth of pathogens [14].

Plants nurture many microorganisms in the phyllosphere and rhizosphere. In 1886, German botanist Anton de Bary, the father of plant pathology, proposed the term endophyte [15]. Endophytes are ubiquitous and reside in the intercellular tissues of about 300,000 plant species without causing any negative impacts [16]. Some predominant prokaryotic and eukaryotic endophytes that have been explored are *Actinobacteria*, *Ascomycota*, *Bacteroidetes*, *Basidiomycota*, *Firmicutes*, *Proteobacteria*, and *Zygomycota* [17]. Endophytes produce several secondary metabolites, enzymes, and hormones that have a vital role in biotechnology. Endophytes′ antagonistic effect and antimicrobial activities prevent the plants from pathogen infection [18]. Thus, the various plant–endophyte interactions have captivated researchers in sustaining the agroindustries. This review encapsulates the fungal- and bacterial-induced stress in plants such as water and nutrient deficiency stress that causes various plant diseases. It also explores the significant role of stress-tolerant fungal and bacterial endophytes, and their biocontrol defense mechanisms such as the production of hydrolytic enzymes, secondary metabolites, siderophores, systemic acquired resistance (SAR), and induced systemic resistance (ISR) in combating fungal and bacterial pathogens.

## 2. Bacterial- and Fungal-Induced Plant Stress

Pathogenic microbes (bacteria and fungi) induce biotic stress in plants. These pathogens invade the plant through the roots, stomata, or open wounds by colonizing the plant xylem vascular bundle, thus occluding the water flow and causing water deficit stress as a result of xylem dysfunctioning in the plant, which ultimately causes vascular disease [5]. Some pathogens use haustorium to deprive the host of nutrients via a biotrophic interface, thus inhibiting the growth of the plant as a result of nutrient deficiency stress and the onset of rust and powdery mildew diseases [19]. Due to this stress, plants experience morphological, physiological, and biochemical variations [7]. Physiological changes affect the roots, xylem, and leaf tissues, resulting in obstructed cell division and alleviated cell elongation in addition to affecting carbon fixation, transpiration, gas exchange reduction, respiration, and the upregulation of defense metabolism genes and downregulation of photosynthesis genes in leaves [20] Morphological changes can reduce the leaf surface area, leaf size, growth of internodes, branching pattern, and root and shoot growth of the plant [7]. Biochemical variation causes an imbalance in hormone regulation and nutrients in plants [7]. Table 1 represents the bacterial- and fungal-induced plant stresses and their diseases.

## 3. Role of Endophytes in Combating Bacterial and Fungal Pathogens

Biocontrol forms an effective substitutional method of plant disease control with a low negative impact on the environment and humans. Biological control is described as the application of beneficial organisms, or by-products, such as phytohormones, metabolites, and enzymes, to alleviate the threats caused by pathogens and aggravate favorable reactions in the plant [14,31]. Endophytes, isolated from plant parts, such as the roots, **shoots**, leaves, flowers, and seeds, have promising potential for development into biocontrol agents (BCAs) to enhance plant growth and development. Most phyto-endophytes exhibit symbiotic, mutualistic, and synergetic interactions within the host plants. This provokes resistance against biotic stress, improves soil fertility, and promotes plant growth. Endophytes are beneficial microbiomes that play a vital role as biocontrol agents and phyto-stimulators [32]. Endophytes stockpile nutrients in plants via the production of siderophores, phosphate, nitrate, and enzymes. They also trigger cellular responses by increasing secondary metabolites and phytohormones such as jasmonic acid (JA), ethylene, and salicylic acid (SA) to build up a robust resistance in plants against pathogens [33]. Thus, the application of endophytic microbes is an environmentally friendly and inexpensive alternative method to combat pathogens [33]

**Figure 1:**
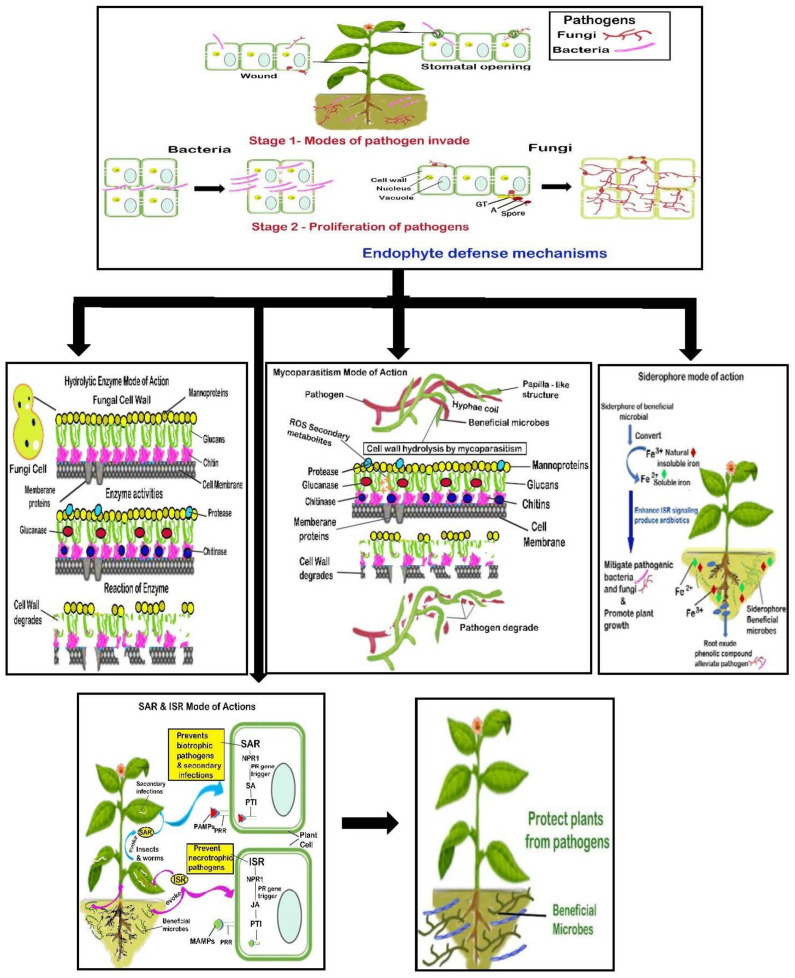
Endophytic biocontrol mode of defense mechanisms against pathogens. Stage 1: Bacteria and fungi invade the plant through a wound or stomatal opening. Stage 2: Both bacteria and fungi degrade the plant cell wall with the aid of enzymes, secondary metabolites, or virulent factors. Bacteria proliferate through the intercellular space, whereas fungi invade the plant by a biotrophic or necrotrophic mechanism using appressorium (A), germ tube (G.T.), and spore and proliferate in the cells of the plant. Endophytes provide defense against pathogens through various modes of action, such as (a) hydrolytic enzyme; (b) mycoparasitism; (c) siderophore; and (d) SAR and ISR modes of action. Note: ISR, induced systemic resistance; JA, jasmonic acid; MAMPs, microbe-associated molecular patterns; NPR1, non-expressor of pathogenesis-related genes 1; PAMPs, pathogen-associated molecular patterns; PRRs, pattern recognition receptors; PTI, pathogen-triggered immunity; SA, salicylic acid; SAR, systemic acquired resistance.

For instance, *Trichoderma* species protect many plants from pathogens by producing inhibiting enzymes, toxic substances, and secondary metabolites and concomitantly promoting plant growth via hormone production [30,34,35]. Endophytes such as *Trichoderma viride*, *Bacillus thuringiensis* SY33.3, *Streptomycetes* spp., and *Pseudomonas fluorescens* suppress the pectinolytic enzyme activities of *Fusarium oxysporum*, which causes vascular wilt in plants [8,34,36]. *Pseudomonas* sp. LBUM300 mitigates *Clavibacter michiganensis*, which causes canker disease in tomatoes by generating antibiotics, namely, hydrogen cyanide (HCN) and 2,4-diacetylphloroglucinol [29]. In general, *Aspergillus* spp., nonpathogenic *Fusarium* spp., *Gliocladium* spp., *Petriella* spp., and *Trichoderma* spp., along with *Bacillus* spp., *Enterobacter* spp., *Lysobacter* spp., *Pantoea* spp., *Pseudomonas* spp., and *Streptomyces* spp., were identified as prime BCAs [17,35,36]. Table 2 and Table 3 **illustrate** the defense mechanisms of fungal and bacterial endophytes against pathogens while Figure 1 depicts the endophytic biocontrol mode of defense mechanisms against pathogens.

Antagonism is the component of a microbial population that suppresses the growth of other microbial communities [31,56]. Hence, microbial community that experience an inhibitory effect can survive. This chemical inhibition is generally known as antibiosis. William Roberts was the first to coin the term antagonism by experimenting with the antagonistic effect between *Penicillium glaucum* and other types of bacteria [31,57]. Hyper-parasitism and antibiosis are the direct antagonist effects of biocontrol that hinder pathogen infections in plants. Antagonistic endophytes are effective BCAs and have a significant role in plant disease management.

### 3.1. Hydrolytic Enzymes

Enzymes are proteins that chemically aid animals and plants to biocatalyze the substrate into a product. This also enhances the plant defense mechanisms to constrain or inhibit biotic stress [42,58,59]. In 1877, Wilhelm Friedrich Kühne was the first to propound the name enzyme, also known as biocatalysts [58]. The microbial enzyme is vital in upgrading plant nutrients, decomposing organic matter, and combating biotic stress. Enzymes have remarkable biotechnological benefits in various fields such as industrial, agricultural, pharmaceutical, and biomedical therapy [17]. Hydrolytic enzymes have an antagonistic property that can inhibit or resist pathogens through the hyper-parasite mechanism and thus have an incredible biocontrol role in crop fertility.

The bacterial cell wall is protected by rigid peptidoglycan or murein, which is lysed by endophytes that produce hydrolytic enzymes such as peptidase, amylase, xylanase, and carboxylase [59]. The fungal cell wall constitutes glycoprotein as an exterior layer, and chitin and β-glucans or α-glucans as an interior layer. Chitin, a chief component of the fungal cell wall, adds rigidity and a skeletal framework to thin cells. α-glucan or β-glucan provides structural rigidity and protects the fungus. Endophytic hydrolytic enzymes can degrade the cell wall of pathogenic fungi and thus protect plants during infection [38,42,60]. For example, chitin synthases (CHSs) trigger the innate immune responses in host plants against fungal pathogen. Since plants are devoid of chitin, endophytes’ CHS enzyme forms an attractive antifungal BCA.

For example, *Trichoderma* species degrades the pathogenic fungal cell wall by producing enzymes such as β-1,3-glucanase, chitinase, N-acetylglucosaminidase, and protease [42] while *Pantoea* and *Curtobacterium* produce enzymes such as protease and endoglucanase [61]. On the other hand, the pistachio causative agent *Paecilomyces formosus* is suppressed by *Streptomyces misionensis* strains (BH4-1 and BH4-3) through hydrolytic enzymes and metabolites [62]. Moreover, *Bacillus halotolerans* FZV 34 and *B. subtilis* FZV-1 can produce metabolites, siderophores, and enzymes. Thus, these endophytes suppress the causative agents that cause root rot infection in pea plants (*Pisum sativum* L.). They also produce antibiotics, namely fengycin and surfactin [63].

However, there is a growing concern about applying live biocontrol microbes in biotechnological applications due to the efficient combatting of specific diseases rather than others. For example, rhizobacteria can only control soilborne diseases but cannot inhibit foliar diseases. Conversely, enzyme-based biofungicides impede pathogens by implying cell-wall-softening enzymes or toxins [58]. Sterilized chitinase isolated from *Serratia* or *Bacillus* sp. showed substantial alleviation in the intensity of citrus fruit rust, rot, and groundnut late leaf spot [64]. Unrivalled utilization of CHS enzymes is procured in biotechnology applications. Hence, microbial-derived enzyme investigation may sustain and reinforce global crop fertility.

### 3.2. Mycoparasitism

The fungi that exhibit parasitic effects on other fungi are mycoparasites [65]. Meagre research has been conducted on biocontrol mechanisms, mainly on *Gliocladium* and *Trichoderma* species [56,66]. Mycoparasite coils around the hyphae or grows adjacent to the virulent fungi and produces the hydrolytic enzyme to degrade the cell wall of the virulent fungi. Mycoparasite interacts with pathogens either as necrotrophs or biotrophs by producing hydrolytic enzymes, antibiotics, or secondary metabolites for antagonistic activity and procuring nutrition from the virulent fungi through a biotrophic interface [65]. For example, *Trichoderma* spp. defend against pathogens via mycoparasitism. In particular, utilization of this genus effectively controls the rhizosphere and phyllosphere phytophthora [34]. *Trichoderma harzianum* and *T. hamatum* show a higher antagonism effect against *Phytophthora capsica,* which influences root rot disease in *Capsicum pubescens*. It was observed that compared to *T. hamatum, T. harzianum* shows a strong mycoparasite effect [56]. Cheong et al. [66] reported that *Diaporthe phaseolorum* (WAA02 and MIF01) and *Trichoderma asperellum* T2 reveal better antagonistic activities against *Ganoderma boninense*, which causes basal stem rot in oil palms. They also pointed out that T2 exhibits mycoparasitic activity while *Diaporthe phaseolorum* (WAA02 and MIF01) shows niche competition.

### 3.3. Niche Competition

Nutrients are the primary source that aids spore germination and regulates the growth of pathogens or endophytes in the host [67]. Biotrophic and necrotrophic pathogens procure specific nutrients from the defected living or dead organisms in the environment [9,68,69]. The presence of exuding nutrients from the wounds, stomatal openings, senescent floral tissues, and dead host tissues of the plants are some of the niche points for the microbes to invade the host [67]. Endophytes occupy such niches and compete with the pathogen by acquiring the essential nutrients and space in the plant, thus preventing the infection of the host [68]. This antagonistic action does not kill the pathogens; instead, it mitigates the pathogenic microbiomes.

Root exudates attract endophytes during stress tolerance. For instance, sugar beetroots combat pathogens by provoking *Flavobacterium* and *Chitinophaga* into the endo-sphere [69]. Likewise, tomato enriches *Flavobacterium* spp. to suppress pathogens [70]. Leguminous plants exude flavonoids during nitrogen starvation to attract N-fixing bacteria [71]. Phyllosphere fungi inhibit rust-induced *Phytophthora infestans* in potatoes through the thigmotropism mechanism, which hinders the availability of stomata for rust spore germination [60]. Landrace maize is enriched with diazotrophic bacteria that facilitate nitrogen fixation [72].

### 3.4. Siderophore Productions

Iron (Fe) is a trace element with redox activities and exhibits a cofactor behavior for many enzymes [73]. Despite being a micronutrient, Fe has essential bioactivities in the growth of living organisms such as enzyme catalyst, electron transfer, DNA and RNA synthesis, and oxygen metabolism [73]. It is insoluble and exists in a ferric hydroxide (Fe (OH)_3_) form. Siderophores are small, high-affinity iron-chelating molecules generated by microorganisms and plants growing in the iron-deficient region. Based on their functional groups, siderophores are categorized into three families, namely, catecholate, hydroxamates, and carboxylates [74]. Siderophores’ function is to accumulate Fe in the cells and inhibit pathogenic organisms. It has an unrivalled affinity with ferric hydroxide and helps organisms to scavenge this element from its surroundings and make it available to the plant cells in a soluble Fe form such as Fe (OH)_2_) [63]. Moreover, it promotes plant growth and thus is involved in various bio-control, bioremediation, chelation agent, and biosensor processes [73].

Siderophores produced by plant growth-promoting bacteria (*Pseudomonas* and *Bacillus* spp.) play a vital role in niche competition by deploying Fe in pathogens and thus mitigating the upshot of pathogens in the plants [63]. Saravanakumar et al. [75] investigated the yeast *Metschnikowia pulcherrima*, which causes the transformation of Fe molecules and pulcherriminic acid into pulcherrimin (red stain), causing an Fe deficiency that retards the growth of pathogens such as *Alternaria alternata*, *Botrytis cinerea*, and *Penicillium expansum* in plants. The in vivo studies of Khan et al. [76] reported that *Allium tuberosum* and endophyte *Acremonium* sp. Ld-03 promotes plant growth by producing siderophore, IAA, and phosphate and protects plants from *Fusarium fujikuroi* and *F. oxysporum*. The co-cultivation of *Streptomyces ciscaucasicus* GS2 and *Cylindrocarpon olidum* triggers a siderophore-mediated defense mechanism and hence amplifies the production of ferrioxamines, which inhibits *Cylindrocarpon destructans, Phytophthora cactorum, Pythium* spp., and *Rhizoctonia solani* AG-5, which cause diseases in apple trees [77].

### 3.5. Secondary Metabolites Productions

Secondary metabolites are bioactive compounds that perform a significant role in defense signaling, ecological interactions, and competition [78]. The establishment of microbial interaction involves the synthesis of secondary metabolites during metabolic exchange, which shows a complex regulatory response. These interactions can be antagonistic, mutualistic, competitive, or parasitic. The latest imaging mass spectrometry (IMS) technology has been used to study mold metabolites and their various functions during microbial interactions [79]. The bioactive secondary metabolites of endophytes have a powerful establishment in the pharmaceutical and agrochemical fields. Bioactive metabolites such as alkaloids, steroids, tannins, terpenoids, quinones, saponins, phenols, and flavonoids produced by endophytes have a prime role in protecting the host from biotic and abiotic stresses [80]. Secondary metabolites have antibacterial and antifungal properties, which control the growth of phytopathogens. Plants can produce secondary metabolites either independently or in association with other endophytes to cope with stress and defense responses during biotic stress [79]. Thus, endophytic secondary metabolites are used as a biocontrol agent to protect plants and improve crop qualities. Plants produce bioactive compounds with insufficient and heterogeneous quality, whereas microbes produce metabolites that are uniform, high quality, and have maximum efficacy regarding their biocontrol potential [80].

Cytochalasin alkaloids are fungal metabolic products with antifungal properties. Hitherto, 300 analogues of cytochalasin have been isolated from *Aspergillus, Chaetomium, Penicillium, Phomopsis, Xylaria*, and so on [81]. *Xylaria* sp. isolated from the leaves of the guarana *Paullinia cupana* plant produces cytochalasin D (1) and piliformic acid (125) metabolites. These metabolites show antifungal activity against *Colletotrichum gloeosporioides*, which causes anthracnose disease in various plants such as citrus, papaya, avocado, eggplant, sweet pepper, and tomato [81]. The fusaric acid derivative (3R,6R)-3-benzyl-6-isopropyl-4-methyl morpholine-2,5-dione (25) obtained from endophyte *Alternaria atrans* MP-7 of *Psidium guajava* exhibited potent antifungal activities against *Alternaria* solani, *Colletotrichum gloeosporioides*, and *Phyricularia grisea* [82]. *Acremonium* sp. Ld-03 shows an antifungal effect against pathogens, namely *Botrytis cinerea, Botryosphaeria dothidea, Fusarium fujikuroi*, and *F. oxysporum*, that infect *Allium tuberosum*. It prevents infections by revealing secondary metabolites such as peptides, xanthurenic acid, cyclic dipeptides, and valyl aspartic acid [76]. *Pseudomonas* strains (*P. donghuensis* 22G5 and *P. protegens* XY2F4) secrete the tropolone compound 7-hydroxytropolone, which has potential resistance against *Verticillium dahlia*, which causes Verticillium wilt in cotton plants [83]. Terpenoids, albaflavenone, β-unsaturated ketone, geosmin, tricyclic α, and 2-methylisoborneol are some volatile odoriferous metabolites distributed in *Streptomyces* that prevent plant infections [84].

### 3.6. Systemic Acquired Resistance (SAR) and Induced Systemic Resistance (ISR)

Plants adapt either local or systemic defense-induced mechanisms to control pathogens, such as systemic acquired resistance (SAR) and induced systemic resistance (ISR) [47,49]. Plants recognize stimuli from causative or non-causative microbes and pests, which ultimately triggers the resistance and priming actions against infective agents. Hence, the plants perceive the pathogen-, microbe- or damage-associated molecular patterns (PAMPs, MAMPs, or DAMPs) through pattern recognition receptors (PRRs). These PRRs trigger a cascade of signals such as pathogen-, microbe-, or effector-triggered immunity (PTI, MTI, or ETI) that boost plant defense against pathogens [33]. Induced defense mechanisms are collateral, which either yields phytoalexins, phenolic compounds, nitrogen oxide, ROS, relocation of nutrients, pathogenesis-related (PR) proteins, antimicrobial metabolites, or build-up of physical barriers such as alteration of cell walls, cuticles, and stomata closure regulation [33].

Systemic acquired resistance (SAR) is a plant resistance response aroused by pathogens and pre-existing pathogen infections [49]. SAR induces local resistance by triggering hypersensitive reaction (HR) via signaling molecules such as salicylic acid (SA) and associated PR proteins to the infected parts and neighboring parts of the plant, thus defending against biotrophic pathogens. SAR acquires long-term protection against a diversity of microorganisms. In 1960, Ross reported that tobacco plants could successfully combat the secondary infections provoked by the tobacco mosaic virus (TMV) in the distal tissue. He termed the proliferation of resistance as SAR [85].

For example, *Bacillus subtilis* induces disease resistance via the SA-dependent signaling pathway, thus controlling *Blumeria graminis f.* sp. *Tritici,* which causes powdery mildew in wheat [86]. Pretreatment of pea (*Pisum sativum*) seeds with *Trichoderma asperellum* (T42) and *Pseudomonas fluorescens* (OKC), which induces the defense response by elevating phytohormone, SA, and PR-1 protein and hinders *Erysiphe pisi*, protects the plant from powdery mildew disease [87]. The pathogenesis-related gene 1 (PR1) is a vital regulator of non-expressor of pathogenesis-related genes 1 (NPR1 and NPR3/4), which ultimately provokes an antagonistic effect via SAR through priming and reveals resistance against secondary infections [88].

ISR is mediated by beneficial microbes living in the rhizosphere [47]. It triggers signaling molecules such as JA and associated PR proteins to the infected parts and leads to plant defense against necrotrophic pathogens. The ISR mechanism does not execute direct killing or inhibit the pathogen. Instead, it augments the physical or chemical barrier of the plants [89]. The ISR signal is unspecified due to the recruitment of varied components by diverse microbes [90]. Generally, JA and its derivative JA-isoleucine (JA-Ile) hormone regulate signaling pathways via abscisic acid (ABA) or ethylene (necrotrophic pathogens defender) [89]. ISR and SAR often show an antagonistic effect, which regulates the cellular-level signaling. Upstream and downstream signaling occurs between SA and JA during the antagonistic effect on necro- or biotrophic pathogens and vice versa [89]. During iron depletion, several signaling molecules, hormones (nitric oxide, auxin, and ethylene), and the transcription factor MYB72 emerge as critical regulators that process and initiate the ISR defense mechanism. Thus, this process enhances the solubility of iron and remodels rhizosphere microbes in the plant. The union of ISR and the iron deficiency response opens the way for the use of ISR-eliciting microbes as iron biofertilizers [91].

*Burkholderia* species (BE17 and BE24) hinder spore germination and mycelium growth of *Botrytis cinerea* via the ISR mode of defense and thus protects grapevine from grey mold disease [92]. *Streptomyces diastato* chromogens KX852460 resist *Rhizoctonia solani* AG-3, which causes tobacco leaf spots via ISR-synthesizing enzymes such as glutathione peroxidase and peroxidase [93]. *Bacillus subtilis* PTA-271 and *Pseudomonas fluorescens* PTA-CT2 act against *Arabidopsis* plant pathogens, namely, *Botrytis cinerea*, which causes grey mold, and *Pseudomonas syringae* Pst DC3000, which causes canker, by inducing ISR and their antagonistic effect is revealed by an increase in the levels of ABA and JA in the leaves of the infected plant [94]. *Trichoderma* spp. AA2 and *Pseudomonas fluorescens* PFS are the most potent inhibitors of *Ralstonia* spp., which causes bacterial wilt in tomatoes by inducing ISR in the plant [95].

Priming enhances plants to sensitize environmental cues without invoking the induction of specific defense genes and accelerates strong responses to biotic and abiotic stresses [96]. Priming is activated via a broad spectrum of mechanisms such as infections with causative agents, colonization of beneficial root microbes, administration of synthetic or natural chemicals, alteration of primary or secondary metabolites, attraction of phenolic compounds, and perception of volatile organic compounds [96]. The resistive reaction of volatile organic compounds (VOCs) increases the emission of aromatic compounds, elevation of oxidative burst, union of hydroxycinnamic acid esters and “lignin-like” polymers inside the cell wall, and multiplied induction of defense genes [96].

Hu et al. [97] discovered that due to the induction of prime signaling, benzoxazinoids, a defensive secondary metabolite, are released from the root of wheat and maize, which alter the root microbes of plants. Thus, they not only increase jasmonate signaling and plant defense mechanism but also suppress the performance of herbivores in the next plant generation.

Schulz-Bohm et al. [98] propounded that exposure of *Carex arenaria* root to the fungal pathogen *Fusarium culmorum* invokes VOCs and attracts the endophytes in the root and suppresses the pathogen. *Burkholderia cenocepacia* ETR-B22 of *Sophora tonkinensis* produces VOCs such as nonanoic acid, benzyl propionate, benzyl acetate, dimethyl trisulfide, methyl anthranilate, methyl salicylate, methyl benzoate, 3,5-di-tert-butyl phenol, and allyl benzyl ether. The bacteria show a wide array of antifungal activities against 12 fungal pathogens, namely *Alternaria alternata, Aspergillus niger, Bipolaris sorokiniana, Botrytis cinerea, Fusarium oxysporum, Fusarium solani, Fusarium fujikuroi, Helminthosporium torulosum, Mycosphaerella fijensis, Magnaporthe oryzae Phyllosticta zingiber*, and *Rhizoctonia solani*, which cause several infections in plants [99].

## 4. Conclusions

Biotic stress influences pathogens and affects the quality of plant growth and productivity. It alters the physiological and biological properties of crops and causes major constraint on crop yield. Fungal and bacterial stresses affect plants and cause diseases that lead to global crop calamity. Beneficial microbes are a capitative biocontrol substitute for pesticides for plant disease management. Endophytes play an active biocontrol role in suppressing pathogens and enhancing crop yields. They protect plants by producing hydrolytic enzymes, secondary antifungal metabolites, and siderophores and considerably improve the antioxidant system. They also induce plant defense via SAR and ISR mechanisms.

This critical review highlights that beneficial endophytic microbes could sustain crop production and resolve the issues regarding crop yield caused by bacterial and fungal pathogens. Based on the current scenario, future research is necessary to identify potential BCAs, which can minimize the utility of synthetic pesticides, increase crop yield, and retain beneficial soil microbes. In order to enhance crop production, meet the demands of a growing global population, and reduce environmental pollution caused by the applications of fungicides and pesticides, it is necessary to significantly increase the production of endophytic BCAs. It is hoped that this review article will provide sufficient information for microbiology researchers on the benefits of using biocontrol endophytes in enhancing crop production. It will also motivate biotechnologists to move forward to commercially produce more BCAs.

## Figures and Tables

**Table 1:** Fungal and bacterial pathogen-induced stress in plants and their diseases.

Pathogen	Mode of Inducing Stress and Plant Disease	References
*Fusarium oxysporum*, *Fusarium oxysporum f.* sp. *pisi*, *F. oxysporum* var. *redolent, F*. *poae*, *F. solani*, and *F. avenaceum*; *Verticillium dahliae*; *Verticillium albo-atrum*	Fungi colonize the xylem vascular bundle then obstruct water flow and induce water stress in fruits and vegetables, which leads to wilt disease.	[5,8]
*Puccinia triticina*, *P. striiformis Westend f.* sp. *tritici*, *P. graminis Pers. f.* sp. *tritici*.	Fungi acquire nutrients from the host via a biotrophic interface and induce a nutrient deficiency in wheat, causing rust disease.	[20]
*Blumeria graminis f.* sp. *hordei*, *Blumeria graminis f.* sp. *tritici*, *Sphaerotheca fuliginea*	Fungi colonize and induce a foliar fungal sink at the infected site and acquire nutrients from the host via an obligate biotrophic interface, producing powdery mildew disease in cereals and grasses.	[9,21]
*Ustilago maydis*	Fungi induce endoglucanase to degrade cellulose and arabinofuranosidase and xylanase to degrade the hemicellulose of the plant cell wall, causing corn smut disease in corn	[22]
*Magnaporthe oryzae*	During fungal infection, increased induction of pectate-lyase, endo-xylanase, cellulase, and hemicellulase enzymes lead to degradation of the plant cell wall, causing rice blast disease in rice.	[23]
*Fusarium verticillioides*, *Fusarium sporotrichioides*	Fungi induce mycotoxins such as fumonisins, T-2 toxin, and trichothecenes, causing kernel, stalk, and ear rot in cereals.	[24]
*Fusicoccum amygdali* Del	Fungi produce fusicoccin, which induces irreversible stomatal opening due to the osmotic swelling of the guard cells, leading to wilting of leaves in peach and almond.	[25]
*Pseudomonas marginalis*	Bacteria induce enzymes that degrade the pectin layers of the plant cells, causing bacterial soft-rot disease in tomato.	[26]
*Erwinia chrysanthemi*	Bacteria induce endo-xylanase activities to degrade plant cellulose, causing stem and root rot in maize.	[27]
*Clavibacter michiganense* subsp. *sepedonicum*, *Ralstonia solanacearum*, *Xanthomonas campestris*, *Clavibacter michiganensis* subsp. *michiganensis* and *Xylella fastidiosa*	Bacteria colonize the xylem vascular bundle, thus occluding water flow and inducing water deficit stress in the plant, leading to ring rot, vascular wilt, bacterial spots, bacterial canker and pierce′s diseases in potatoes, tomatoes, pepper, and grapevine, respectively.	[28,29,30]
*Pseudomonas syringae*, Pv. *syringae*	Syringomycin E and G and syringopeptin 25A toxin induced by bacteria, which inhibit plant growth, affect H^+^-ATPase activity, and induce electrolyte leakage in plant tissues, causing bacterial canker in carrot, potato, and tobacco.	[25]

**Table 2:** Defense mechanism of fungal endophytes against pathogens.

Fungal Endophyte	Mode of Defense Actions against Pathogens	References
*Trichoderma viride* and *T. harzianum*	Endophytic curbing of *Fusarium verticillioides* and *F. proliferatum*, which cause stalk rot disease in maize, by producing antifungal acetonic extracts of acetic acid and palmitic acid, and showing mycelial growth. Mycoparasitism mode of antagonistic activities is only observed in *T. viride*.	[34]
*Trichoderma harzianum*	Endophyte produces antifungal metabolites and controls *Ralstonia solanacearum*, which causes bacterial wilt in tomato.	[30]
*Simplicillium lanosoniveum*	Endophyte exhibits a mycoparasite mode of antagonistic activity against *Phakopsora pachyrhizi*, which causes rust in soybean.	[37]
Arbuscular mycorrhizal fungi (AMF)	Endophytes combat *Phoma medicaginis*, which causes alfalfa leaf spots in alfalfa, by inducing defense activity, including jasmonic acid (JA), salicylic acid (SA), peroxidase (POD), and polyphenol oxidase (PPO).	[38]
*Trichoderma harzianum* LTR-2	Endophyte exhibits a drastic reduction in proliferation of the causative agent *Plasmodiaphora brassicae*, which causes clubroot in cabbage.	[39]
*Aureobasidium* strains *A. pullulans, A. subglaciale* and *A. melanogenum*	Endophytes defend *Botrytis cinerea*, which causes grey mold in tomato and grapes via 3-methyl-1-butanol volatile organic compound (VOC).	[40]
*Trichoderma asperellum* T1 and *Trichoderma spirale* T76-1	Endophytes control *Corynespora cassiicola* and *Curvularia aeria* which cause leaf spot disease in lettuce by producing extracellular enzymes such as chitinase, POD, β-1,3-glucanase, and PPO.	[35]
*Rhizobium Vitis* ARK-1	Endophyte shows an antagonistic effect by producing antibiosis against virulence genes virA, virD3, and virG of *Rhizobium Vitis* (Ti), which causes crown gall in grapevine.	[41]
*Trichoderma asperellum* PQ34	Endophyte produces chitinase enzymes that significantly reduce the prevalence of *Sclerotium rolfsii* and *Colletotrichum* sp., which causes anthracnose in peanut.	[42]
*Trichoderma virens* FY06	Rhinomilisin B (41), divirensol H (42), and trivirensol A (43) produced by endophytes exhibit potent antifungal activity against *Colletotrichum gloeosporioides*, which causes anthracnose in lychee.	[43]

**Table 3:** Defense mechanism of bacterial endophytes against pathogens.

Bacterial Endophyte	Mode of Defense Actions against Pathogens	References
*Bacillus subtilis XZ18-3*	Endophyte shows antagonistic antifungal activity by accumulating reactive oxygen species (ROS) in *Rhizoctonia cerealis*, which causes rot and blight in wheat.	[44]
*Bacillus thuringiensis* SY33.3	Endophyte shows an antagonistic effect by producing an extracellular chitinase enzyme against causative agents such as *Fusarium oxysporum f.* sp. *niveum*, *Verticillium dahlia*, and *Aspergillus niger,* which causes wilt and black mold in grapes, apricots, onions, and peanuts.	[8]
*Streptomyces roseoverticillatus* 63 (Sr-63)	Endophyte shows an antagonistic effect by producing carbazomycin B metabolite against the pathogen *Xanthomonas oryzae* pv. *oryzae*, which causes bacterial leaf blight in rice. It inhibits the pathogen metabolic activity by decreasing the malate dehydrogenase activity and suppressing the pathogen protein expression.	[45]
*Bacillus velezensis* Bs006	Endophyte shows antagonistic activity by producing antimicrobial cyclic lipopeptides such as turins, surfactants, and fengycins and suppresses *Fusarium oxysporum f.* sp. *Physalis*, which causes *Fusarium* wilt in goldenberry.	[46]
*Bacillus circulans* GN03	Endophyte isolated from *Brassica Chinensis* has unrivalled efficacy in plant growth promotion and disease resistance by a significant hoard of defense and growth-related hormones (SA, JA, gibberellic acid, brassinosteroid, and indole-3-acetic acid (IAA)). The in vivo model shows that endophytes protect cotton seedlings against the *Verticillium dahliae* strain V991, whichcauses *Verticillium* wilt.	[47]
*Chromobacterium vaccinii*; *C. vaccinii* MWU328, MWU300, and MWU205	Endophytic strains have a broad spectrum of antifungal VOC activity that mitigates the growth of the *Phoma* sp. and *Coleophoma* sp., which cause fruit rot in cranberry.	[48]
*Bacillus* sp. strains G4L1	Endophyte actively resists *Ralstonia solanacearum*, which causes bacterial wilt in tomato, via upregulation of the lipoxygenase gene in the stem, expression of the PR-1 gene and Glutelin genes in roots, and protection of the plant by induction of JA, SA, and the ethylene-dependent defense signaling pathway.	[49]
*Bacillus velezensis* strain J.K.	Endophyte isolated from the rice hybrid variety *Oryza sativa* L., Shenliangyou 5814 produces secondary metabolites that show a significant antagonistic effect against the pathogen *Magnaporthe oryzae*, which causes rice blast in rice.	[50]
*Pantoea dispersa* (RO-18, RO-20, RO-21, and SO-13)	Endophytes strongly inhibit mycelium growth and spore germination and modify the morphology of *Ceratocytis Fimbriata* hyphae via antifungal effects and curb black rot in sweet potato.	[51]
*Streptomycetes* sp. strain FJAT-31547	Endophyte shows an antagonistic effect by producing the antifungal component *n*-hexadecanoic acid against the growth of *Fusarium oxysporum* and *Ralstonia solanacearum*, which causes *Fusarium* wilt and bacterial wilt in tomato, respectively.	[36]
*Aspergillus capensis* CanS-34A	Endophyte isolated from *Brassica napus* produces antifungal the metabolite rosellichalasin (3), which inhibits phytopathogens such as *Botrytis cinerea*, *Monilinia fructicola*, *Sclerotinia sclerotiorum*, and *S. trifoliorum*, which cause grey mold, rot and canker, and rot in fruits and vegetables, respectively.	[52]
*Streptomyces angustmyceticus* NR8-2	Endophyte produces β-1, 3-glucanase antifungal metabolites and volatile compounds to suppress *Curvularia lunata* and *Colletotrichum* sp., which cause leaf spots in *Brassica rapa* subsp. *Pekinensis.*	[53]
*Streptomyces* AMA49	Endophyte produces antifungal metabolites oligomycin A and its derivatives, which show an antagonism effect by inhibiting the germination of conidia and formation of appressorium of the pathogen *Pyricularia –* *oryzae*, which causes rice blast in rice.	[54]
*Pseudomonas aeruginosa*, *Burkholderia gladioli*, *Burkholderia rinojensis*, and *Burkholderia arboris*	In vitro and in vivo studies revealed that these endophytes have a significate antifungal antagonistic effect against the seed colonization pathogen *Colletotrichum truncatum*, which causes anthracnose in pepper.	[55]

## Data Availability

Study does not involve any huge data acquisition and the corresponding author may be contacted for further assistance of the subject discussed.

## Sample Availability

Not available.
